# Recent advances in nanoarchitectures of monocrystalline coordination polymers through confined assembly

**DOI:** 10.3762/bjnano.13.67

**Published:** 2022-08-12

**Authors:** Lingling Xia, Qinyue Wang, Ming Hu

**Affiliations:** 1 Engineering Research Center for Nanophotonics and Advanced Instrument (MOE), School of Physics and Electronic Science, East China Normal University, Shanghai 200241, China

**Keywords:** applications, assembly, coordination polymer, metal-organic frameworks, nanoarchitectonics

## Abstract

Various kinds of monocrystalline coordination polymers are available thanks to the rapid development of related synthetic strategies. The intrinsic properties of coordination polymers have been carefully investigated on the basis of the available monocrystalline samples. Regarding the great potential of coordination polymers in various fields, it becomes important to tailor the properties of coordination polymers to meet practical requirements, which sometimes cannot be achieved through molecular/crystal engineering. Nanoarchitectonics offer unique opportunities to manipulate the properties of materials through integration of the monocrystalline building blocks with other components. Recently, nanoarchitectonics has started to play a significant role in the field of coordination polymers. In this short review, we summarize recent advances in nanoarchitectures based on monocrystalline coordination polymers that are formed through confined assembly. We first discuss the crystallization of coordination polymer single crystals inside confined liquid networks or on substrates through assembly of nodes and ligands. Then, we discuss assembly of preformed coordination polymer single crystals inside confined liquid networks or on substrates. In each part, we discuss the properties of the coordination polymer single crystals as well as their performance in energy, environmental, and biomedical applications.

## Introduction

Coordination polymers are hybrid materials with infinite structures formed by assembly of metal or metal-based clusters and molecular ligands. The structures of the coordination polymers are generally determined by analyzing single crystals [1–4]. With the development of suitable synthetic strategies, various kinds of monocrystalline coordination polymers have become available [5–7]. Investigation of these monocrystalline coordination polymers not only provides structural information but also discloses physical and chemical properties [5–7]. With the discovery of interesting properties such as ultrahigh porosity, uniform porous structures, or defined catalytic centers, monocrystalline coordination polymers have become an emerging class of materials with great promise in a great number of applications [8–12]. Because of the broad interest from various fields, there is a great need for tailoring the properties of monocrystalline coordination polymers. Although coordination polymers are naturally tailorable by changing nodes and ligands of coordination polymers through molecular or crystal engineering, the practical needs sometimes require the properties to exceed those of the coordination polymers themselves [4,13–18]. Therefore, novel strategies are highly needed.

Nanoarchitectonics is a concept that overlaps greatly with molecular engineering and nanotechnology [19–27]. The assembly of micrometer-sized, nanometer-sized and/or molecular building blocks is a central part of nanoarchitectonics [19–27]. This is very useful in controlling the formation and properties of monocrystalline materials [28–46]. In particular, when assembly occurs in confined environments, new opportunities arise for changing the properties of materials.

Assembling ionic/molecular building blocks in a liquid that contains impurities generally does not change the intrinsic crystal structure of the obtained single crystals. However, the crystallization kinetics can be affected, which eventually leads to nanoarchitectures [28–46]. For instance, faster growth can be realized in polyelectrolyte solution with the help of shear flow [28]. The crystal fronts move very fast. Thus, the networks hindering movement of the crystal fronts may be encapsulated in the single crystals [29–38]. When the encapsulated networks are removed by etching or dissolving, the previously occupied spaces can be freed, leaving periodic or random mesospace inside the single crystals [29–38]. The networks can also help to take other components into the single crystals. Nanoparticles, such as quantum dots or iron oxide, which attach to the networks, can be encapsulated within the networks inside the single crystals, rendering the single crystals fluorescent or magnetic [39–41]. The networks themselves can also change the properties of the single crystals [42–46]. For example, commonly known fragile and rigid single crystals become soft by integrating polymer networks [45]. The thermal stability of some single crystals may also be improved by incorporating polymeric scaffolds [46].

Confined assembly of preformed monocrystalline materials and other microscopic building blocks through specific interactions or in limited spaces is efficient in packing the materials [47–55]. Interesting properties may be brought by forming specific alignments. For instance, monodispersed colloidal particles can assemble through the evaporation of droplets [56–57]. The local flow and equilibrium interactions among the particles force the obtained assemblies to have periodic structures. The ordered colloidal crystals can regulate the transmission/reflection of photons [57–58]. Assembly of nanometer-sized monocrystalline materials with anisotropic shapes is of particular importance because the interactions between the crystals can be stronger than among quasi isotropic crystals [59–62]. However, spontaneous assemblies of anisotropic crystals are less periodic than those of isotropic crystals, because the movement of 1D or 2D crystals during evaporation of the droplets is often hindered due to steric effects. Confined assembly helps to optimize the alignment of 1D or 2D crystals by introducing additional flow, that is, shear flow or Marangoni flow [63–69]. Confined assemblies of 1D or 2D crystals are tough and deformable in contrast to the fragile nature of the building blocks [63–69].

In recent years, confined assembly has been introduced into the field of coordination polymers. It has been significant for tailoring the properties of or introducing new functions to coordination polymers [70–93]. In this review, we will discuss crystallization of monocrystalline coordination polymers in confined environments, such as confined liquid networks or substrates. Then, we will discuss the assembly of the preformed monocrystalline coordination polymers inside confined liquid networks or on substrates. In both parts, the change of the properties of the coordination polymers will be summarized. The performance of the obtained materials will be evaluated regarding advanced applications in energy, environment, and biology.

## Review

### Confined crystallization of monocrystalline coordination polymers

**Figure 1:**
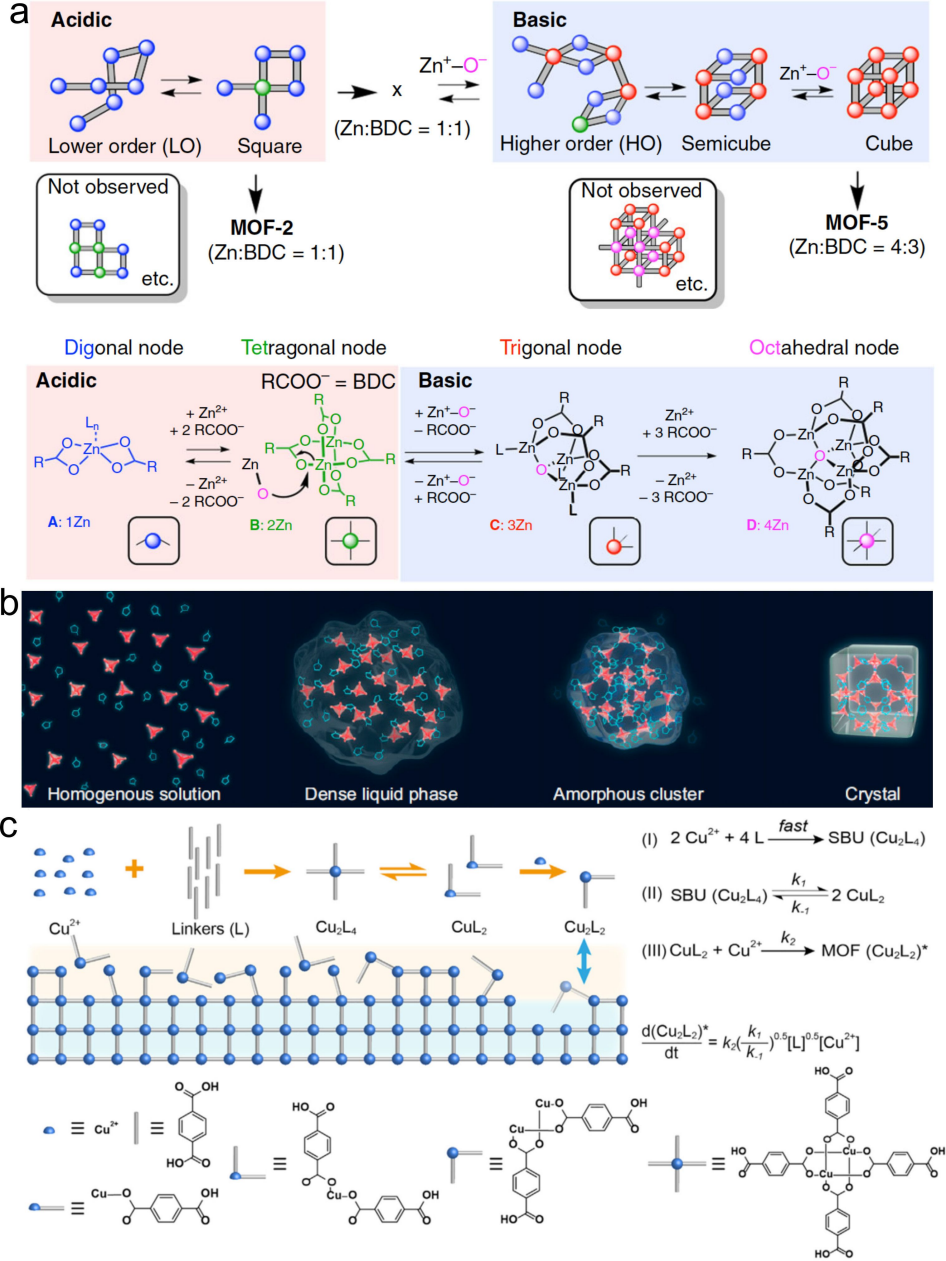
(a) Schematic illustration of the formation paths of MOF-2 and MOF-5 crystals. Low-order (LO) and high-order (HO) PNCs were generated under, respectively, acidic or basic conditions. Digonal, trigonal, tetragonal, and octahedral nodes are shown. Figure 1a was reproduced from [95] (© 2019 J. Xing et al., published by Springer Nature, distributed under the terms of the Creative Attribution 4.0 International License, https://creativecommons.org/licenses/by/4.0). (b) Schematic illustration of the nucleation process of ZIF-8 with multiple transition phases. Figure 1b was reproduced from [96]. According to the PNAS license conditions only noncommercial use is allowed. This content is not subject to CC BY 4.0. (c) Mechanism for the growth of Cu-MOF-2 single crystals involving the generation of Cu_2_L_4_ SBU and its fragmentation into CuL_2_. Figure 1c was reprinted from [97], Chem, vol. 8, by J. Han et al., “Determining factors in the growth of MOF single crystals unveiled by in situ interface imaging”, pages 1637-1657, Copyright (2022), with permission from Elsevier. This content is not subject to CC BY 4.0.

Crystallization of single crystals basically involves nucleation and growth stages. During both stages, the assembly of atomic, ionic, or molecular building blocks is governed by thermodynamic and kinetic rules. Despite the fact that the main crystallization processes of coordination polymers are governed by similar rules, the detailed mechanism can be different owing to the complex topology, coordination bonds, and secondary building blocks of coordination polymers [94]. Ex situ and in situ characterizations and computer simulations have been employed to investigate and analyze the crystallization processes of several coordination polymers [94–97]. The results suggest that there are many kinds of prenucleation clusters (PNCs), transition phases, and actual second building units (SBUs) in the prenucleation and growth steps (Figure 1). Transition and attachment of these species are not only controlled by thermodynamics but also strongly depend on kinetics. The decisive factors involve diffusion, local flow, and gradient concentration of modulators, which are highly tunable in a confined environment [98–101]. Therefore, crystallization of the monocrystalline coordination polymers in confined environments can be of great significance [98–101].

Microfluidic systems offer controlled channels with defined structures and flow characteristics. Thus, they are good experimental tools for the observation of crystallization process. The interfaces among the laminar fluids in microfluidic channels can be recognized as soft boundaries of crystallization zones. In the reaction–diffusion zone confined by liquid–liquid interfaces (soft boundary), crystallization of a 2D coordination polymer ([Cu(4,4′-bpy)](NO_3_)_2_, 4,4′-bpy = 4,4′-bipyridine) was investigated [102]. By changing the flow rate ratio between the focusing streams and the reagent fluids, the size and concentration gradient of the reaction–diffusion zone could be defined, leading to diffusion-limited and kinetically controlled environments. Nonequilibrium crystal morphologies were observed. Needles were found to assemble into frames, which were subsequently woven into plate-like single crystals.

**Figure 2:**
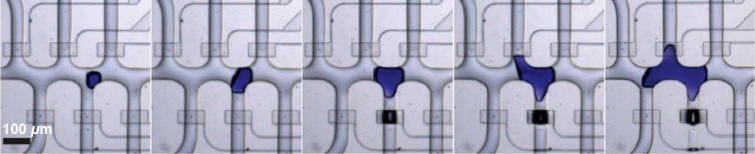
Bright-field images taken during the confined growth of a branched CuGHG single crystal within a microfluidic device. The crystal is of purple color. Figure 2 was reprinted with permission from [103], Copyright 2020 American Chemical Society. This content is not subject to CC BY 4.0.

The channel walls of microfluidic reactors are hard boundaries for crystallization. The diameters of the channels are usually in a range of several tens or hundreds of micrometers, which matches the sizes of bulk coordination polymers. Thus, when the coordination polymer crystals are large enough, their fronts can touch the channel wall during growth. However, the hard boundary cannot be deformed or passed through, changing the shape of the crystal far from the intrinsic symmetry. A type of peptide-based metal-organic frameworks (CuGHG, GHG = glycine–ʟ-histidine–glycine) was grown in microfluidic channels [103]. Several branches of the connected channels were fully occupied by one single crystal (Figure 2). The fronts of the single crystal grew along these channels, leading to single crystals with several branches, such as comb-shaped single crystals, as investigated by optical microscopy. It was found that when the crystal size is smaller than the channel size, the crystals were polyhedra. When the channel was fully filled by a crystal, chemical gradient, physical constraints, and absence of advection changed the properties of the crystal according to reaction–diffusion theory.

The fact that one single crystal can occupy several connected branches suggests that the channel walls can be encapsulated by single crystals. This is a basis for the formation of composites with a single crystal matrix. When solid networks are immersed into a solution of coordination polymers, the connected space among the networks can be seen as connected vessels to be filled with liquid. When the coordination polymers nucleate and grow inside the connected vessels, the front of the crystals may swallow the networks under fast crystallization kinetics. Therefore, single crystals with encapsulated networks can be formed [41–42].

**Figure 3:**
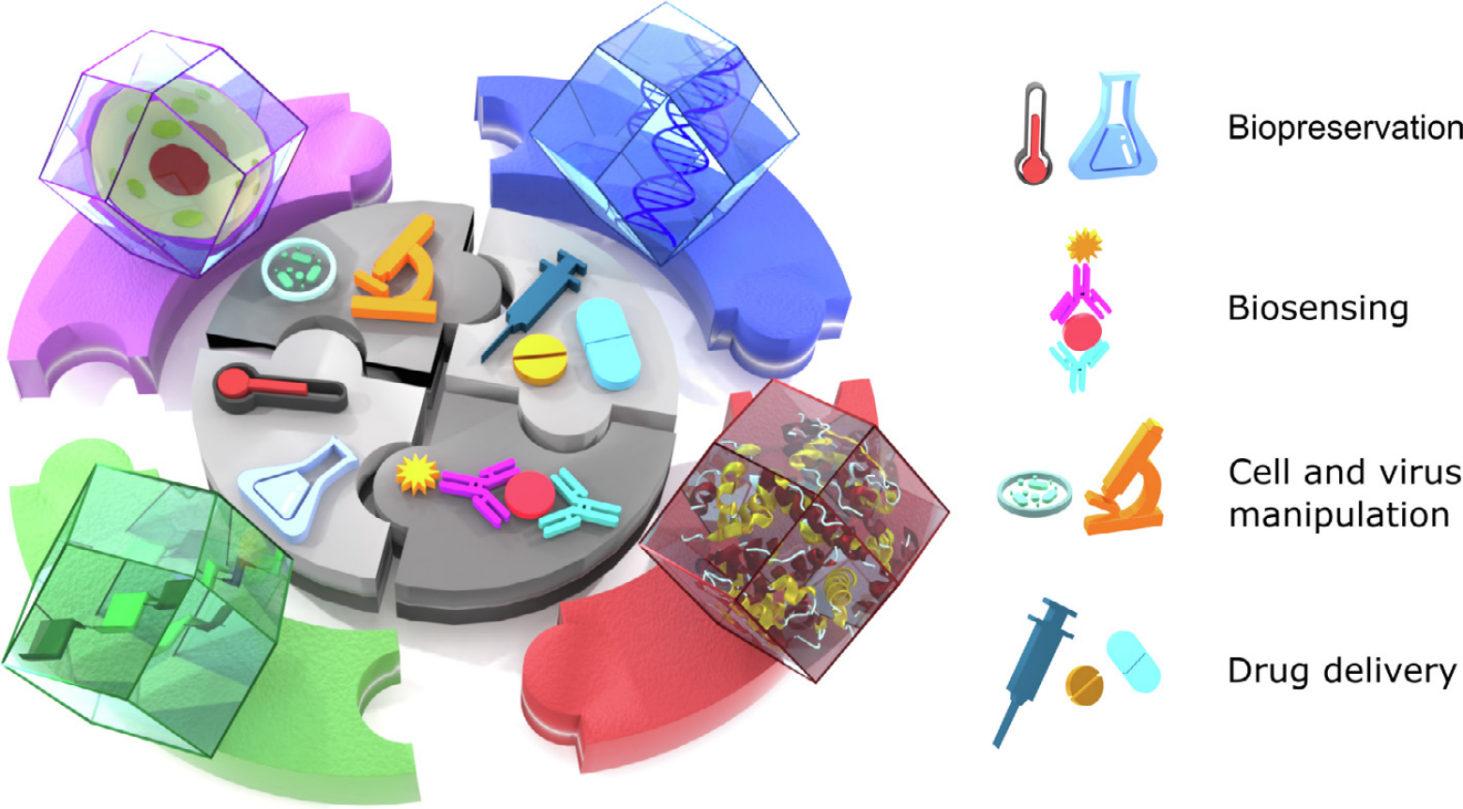
Monocrystalline coordination polymers encapsulated with bioentities and their applications in nanomedicine, biospecimen preservation, biosensing, and cell and virus manipulation. Figure 3 was reproduced from [105] (© 2021 M. D. Velásquez-Hernández et al., published by Elsevier, distributed under the terms of the Creative Commons Attribution 4.0 International License, https://creativecommons.org/licenses/by/4.0).

This strategy offers a great opportunity to store bio-entities into coordination polymer single crystals. Bio-entities, such as enzymes, can form networks in solution. They can attract nodes and ligands to trigger the formation of coordination polymers. Crystallization happens around the networks, rendering monocrystalline coordination polymers as envelopes for the bio-entities. On the basis of the sensitive coordination bonds, the stored bio-entities may be released from solution triggered by pH value or other factors. In 2015, Falcaro et al. demonstrated that monocrystalline ZIF-8 can take up proteins, enzymes, and DNA [104]. The negatively charged biomacromolecules were believed to be one of the key factors to guide nucleation and growth of ZIF-8 crystals. The monocrystalline shells provide specific protection for the relatively weak biomacromolecules. Therefore, the protected biomacromolecules can remain active under harsher conditions, such as higher temperatures, and can be released as needed. This finding shows great potential in applications such as nanomedicine, biospecimen preservation, biosensing, and cell and virus manipulation. The related reports have been nicely summarized in a recent review (Figure 3) [105]. There are several parameters of the single crystals, such as defects, hydrophilicity, and dispersity in water, that determine the performance of the composites in biomedicine [106–107]. To introduce more controllable and repeatable synthetic methods to tailor the parameters of monocrystalline coordination polymer shells, flow syntheses have been used recently [107–108].

**Figure 4:**
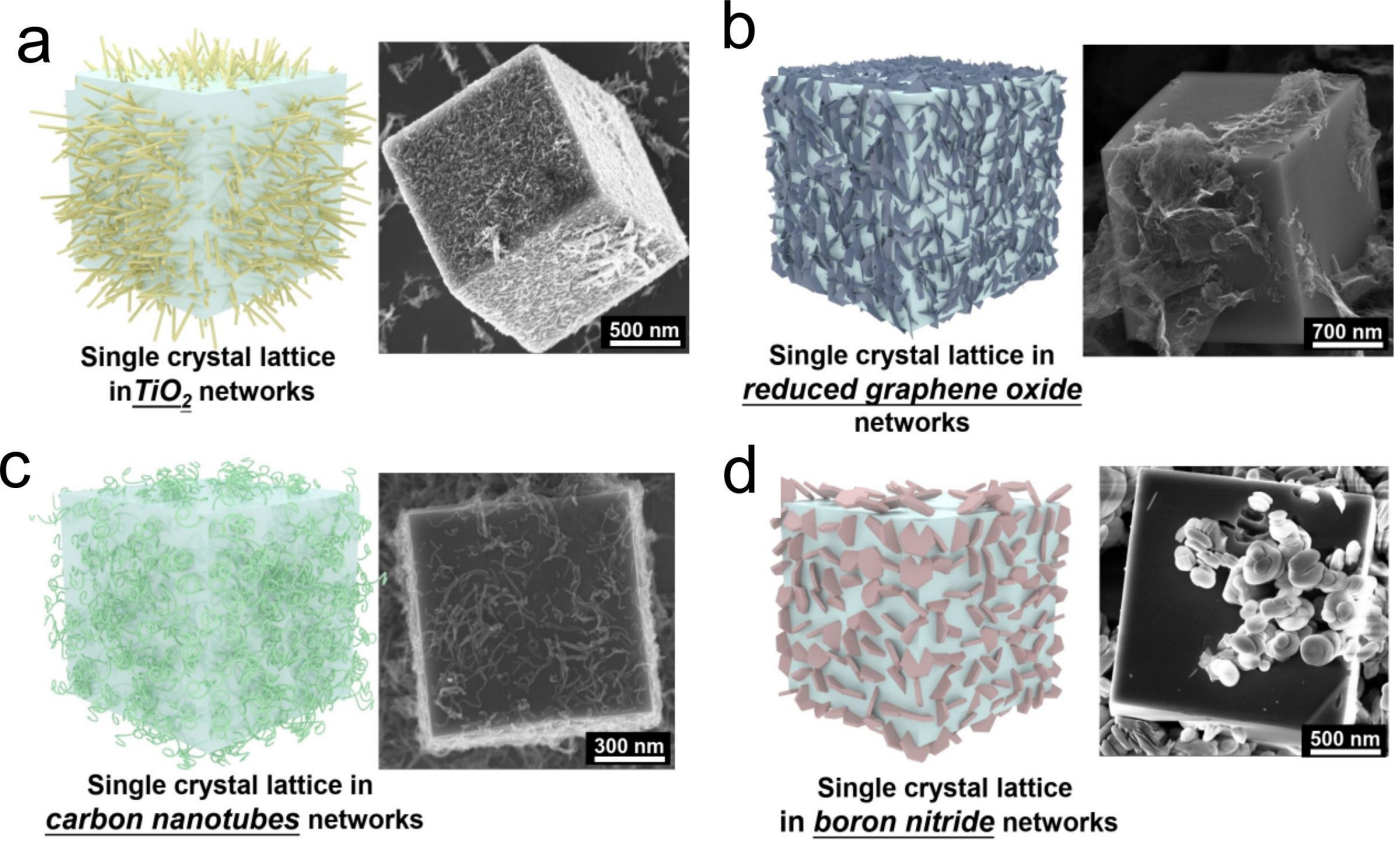
Schematic illustrations and SEM images of Prussian blue single crystals encapsulating various kinds of networks: (a) TiO_2_ networks, (b) reduced graphene oxide networks, (c) carbon nanotubes, and (d) boron nitride networks. Figure 4 was reprinted with permission from [111], Copyright 2021 American Chemical Society. This content is not subject to CC BY 4.0.

The encapsulation of networks can help to regulate the properties of monocrystalline coordination polymers. Bare or modified carbon nanotubes could be modified to allow coordination polymers to grow around [109–110]. This is used to form monocrystalline coordination polymers embedding a fast electron transfer route [110]. The mixed ion-electron of Prussian blue crystals could be significantly enhanced under low temperature (i.e., −20 °C), which is important for the use of batteries in cold regions. To encapsulate conductive networks into Prussian blue single crystals, a gradient crystallization environment was built up through using a volatile inhibiting agent [111]. Evaporation of the inhibiting agent naturally forms a vertical gradient in the reactor, forcing nucleation and growth of single crystals inside the network at the bottom of the reactor. Through this strategy, various networks with different compositions could be encapsulated into monocrystalline coordination polymers (Figure 4) [111]. The rate performance and cycling stability of sodium ion batteries, potassium ion batteries, and seawater batteries were increased by using these materials as positive electrodes. The networks can also help to accelerate ion transfer in coordination polymers. When the Prussian blue nanocrystals contain a double-network PAAm/PAMPS hydrogel, the uptake of Cs^+^ ions from solution could be as high as 397 mg·g^−1^, which is very attractive for purification of seawater contaminated by ^137^Cs [112].

Networks can capture discrete molecules or nanoparticles. When networks are encapsulated into single crystals, the captured molecules or nanoparticles can be co-encapsulated to provide additional functions to single crystals [39–41]. This is applicable to monocrystalline coordination polymers as well. For instance, poly(ethyleneglycol) (PEG) molecular chains show an affinity to Zn^2+^ ions, and can, thus, trigger mineralization of ZIF-8 crystals around. Doxorubicin, a kind of antitumor drug, could be dissolved in the parent solution of ZIF-8 in the presence of PEG chains [113]. When ZIF-8 started to crystallize around the PEG chains, the doxorubicin could be co-encapsulated into the ZIF-8 crystals. The encapsulated doxorubicin could be delivered intracellularly by taking advantage of the controlled decomposition of ZIF-8 under acidic environment. Enhanced cell toxicity was found by encapsulating the doxorubicin drug into ZIF-8 crystals because intracellular uptake was facilitated. When multiple cargos were required, this strategy showed good feasibility. Multiple molecules could be simultaneously encapsulated during formation of single crystals around present networks. One important application is the assembly of vaccine particles. For instance, antigen (ovalbumin, OVA) and adjuvant (cytosinephosphate-guanine oligodeoxynucleotides, CpG) could be incorporated into ZIF-8 nanocrystals with the help of PEG networks [114]. The assembled vaccine particles showed improved immunogenicity. Moreover, the activity of the encapsulated OVA and CpG was maintained under enzyme incubation, heating treatment (60 °C for 12 h), or long-term storage (longer than six months) at 25 °C. The efficiency of this strategy encourages large-scale production. Automation of such an assembly of vaccine particles was realized by using a flow chemical process [115]. By connecting with computer networks, remote synthesis of vaccine particles also became possible.

**Figure 5:**
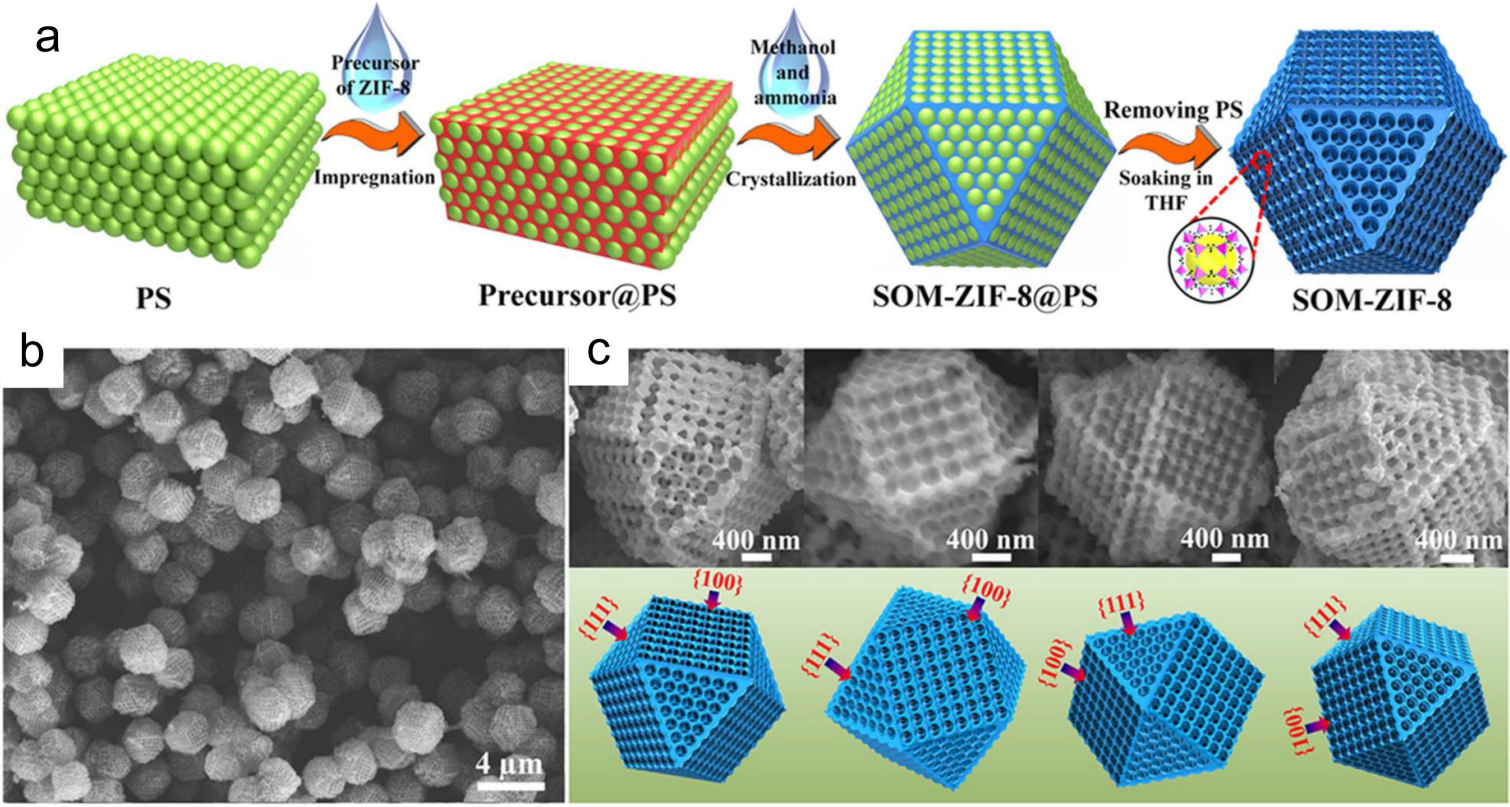
(a) Schematic illustration of synthesis of SOM-ZIF-8. SOM stands for single-crystal ordered macropore. THF is tetrahydrofuran. (b) SEM image of SOM-ZIF-8. (c) Representative SEM images of SOM-ZIF-8. Figure 5 is from [117]. Reprinted with permission from AAAS. This content is not subject to CC BY 4.0.

In some cases, the encapsulated networks in monocrystalline coordination polymers could be selectively removed, for instance, by using appropriate solvents, forming porous textures in the monocrystalline coordination polymers. This feature can solve the intrinsic problem of microporosity in coordination polymers. The micropores of the coordination polymers offer ultrahigh specific surface areas, which are important for increase the density of active sites for catalysis and energy storage. However, they are too narrow for the transport of small molecules, hindering fast mass transport. Forming additional porous textures in monocrystalline coordination polymers can perfectly solve this problem by creating meso–macro channels without destroying most of the micropores. This can be achieved by removing emulsion networks from the single crystal [116]. The obtained HKUST-1 crystals contained bimodal or trimodal pores, which facilitated adsorption and enabled a fast Friedländer reaction. When the networks were formed by densely packed monodispersed polystyrene spheres, ordered macropores could be created inside ZIF-8 single crystals or other coordination polymers (Figure 5) [117–118]. The inverse opal single crystals presented higher catalytic activity than microporous single crystals. These single crystals could also serve as templates to derive microporous CoSe_2_@C, which was used as cathode for aluminum-ion batteries [119]. The connected macropores could facilitate the diffusion of large chloroaluminate anions and provide more exposed active sites, thus, showing excellent rate capacity. Such an enhancement could also be found in the case of electrodes for supercapacitors. The carbons derived from the inverse opal single crystals showed excellent cycling stability [120].

When the networks do not have lattice similarity with the grown monocrystalline coordination polymers, the crystal structure or orientation of the monocrystalline coordination polymers are hardly affected. However, when solids have a similar lattice structure with the grown coordination polymers, epitaxy happens to guide the growth of the coordination polymers. The crystallographic orientation of the grown coordination polymer matches with the surface of the substrate, triggering isotropic or preferential growth of the monocrystalline coordination polymers [121]. Epitaxial growth is significant for future application of the coordination polymers, particularly for electronic applications [122]. The key prerequisite is to find appropriate substrates with lattice similarity to the preferred crystallographic orientation of the targeted coordination polymer. Parallelly aligned Cu(OH)_2_ nanobelts were used to guide the epitaxial growth of Cu_2_(BDC)_2_ (BDC = benzenedicarboxylic acid). Centimeter-scale micropore alignment was realized, showing an anisotropic alignment of fluorescent guest molecules and directional fluorescence [122]. To match with the lattice structure, the grown coordination polymer sometimes may rotate to find the best lattice match with the substrate [123]. In most cases, the lattices of the substrate and the grown coordination polymers can be distinguished by X-ray diffraction analysis [124–127]. However, the flexibility of the frameworks brought unexpected phenomena [128–129]. The shell crystal could first yield to the core crystal, making the whole composite present a single phase as the core crystal [129]. After growing thicker, the interfacial stress forced the core crystal to yield to the shell crystal, making the whole composite present a single phase as the shell crystal [129]. The grown monocrystalline coordination polymer layer dominated the electrochemical behavior of the whole composite in energy storage [129]. The connected core and shell frameworks presented a Na^+^ ion intercalation behavior governed by the outermost layer.

### Confined assembly of monocrystalline coordination polymers

Monodispersed monocrystalline coordination polymers are building blocks for superstructures through self-assembly [130]. The assembly process is usually driven by thermodynamics to form entropy-favored periodic arrangements. The periodically assembled monocrystalline coordination polymers have unique features. For instance, the assembly of polyhedral coordination polymers can form a more complex packing than spherical particles. In addition, their porous structure can accommodate guest molecules, which can further alternate the properties of the whole assembly. These features give the assembled superstructures more freedom to change their properties, such as the photonic bandgap, which has been demonstrated in millimeter-sized superstructures formed by ZIF-8 or UiO-66 [131]. Owing to the porous structure of the monocrystalline coordination polymers. The dielectric constant of the particles may be changed upon adsorption of molecules such as organic vapor. This can lead to a change of the structural color of the assembled superstructure, making optical sensors possible [132–133].

**Figure 6:**
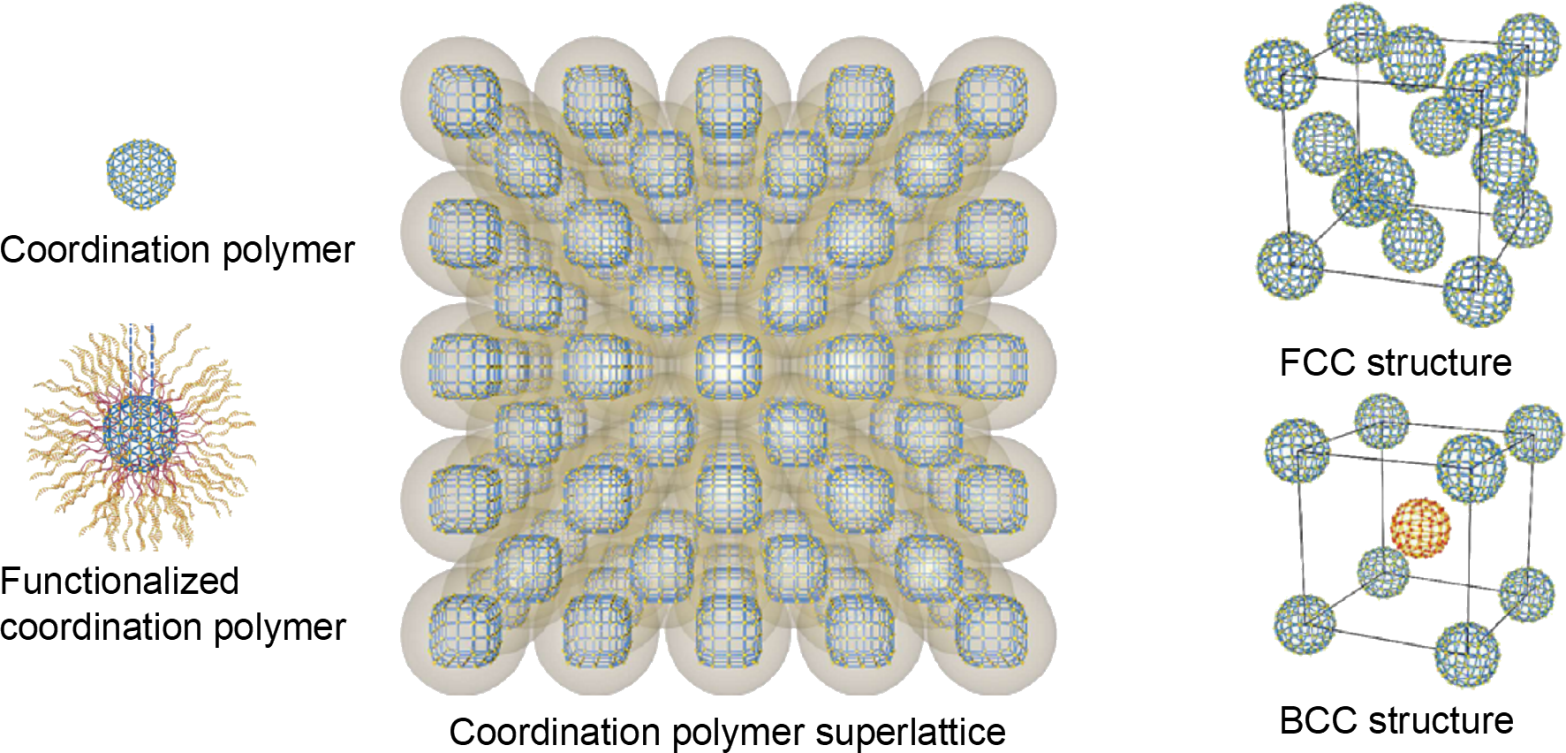
Schematic illustration of a superlattice assembled by DNA-functionalized UiO-66 nanoparticles. Figure 6 was adapted from [134] (© 2020 S. Wang et al., published by Springer Nature, distributed under the terms of the Creative Commons Attribution 4.0 International License, https://creativecommons.org/licenses/by/4.0).

To realize more possibilities of controlling the assembled superstructures, several strategies have been employed. Changing the interparticle interactions through molecular modification is one of the most employed strategies. With surface modifications, the interparticle interactions can be stronger and well defined. With the assistance of interactions among the oligonucleotides, the packing of coordination polymers could be programmed (Figure 6) [134]. 2D and 3D structures, that is, face-centered cubic, body-centered cubic, and CsCl structures were all available [134]. In particular, the 2D assembly showed significant activity regarding the photooxidation of mustard gas [134]. The specific site of the coordination polymer crystals could be modified to generate patchy colloids. By selectively growing a thin layer of UiO-66 on two facets of MIL-96 microcrystals, patchy colloidal particles could be synthesized [135]. The UiO-66 parts show an affinity to oligomers of 3-(trimethoxysilyl)propyl methacrylate (*o*-TPM). The *o*-TPM worked as glue to assemble the patchy particles into supra-chains as needed. The patchy particles could be extended into more complex clusters. Each of exposed facets of the polyhedral coordination polymer crystals could be attached with one polystyrene sphere, forming clusters with various coordination numbers and geometries [136].

**Figure 7:**
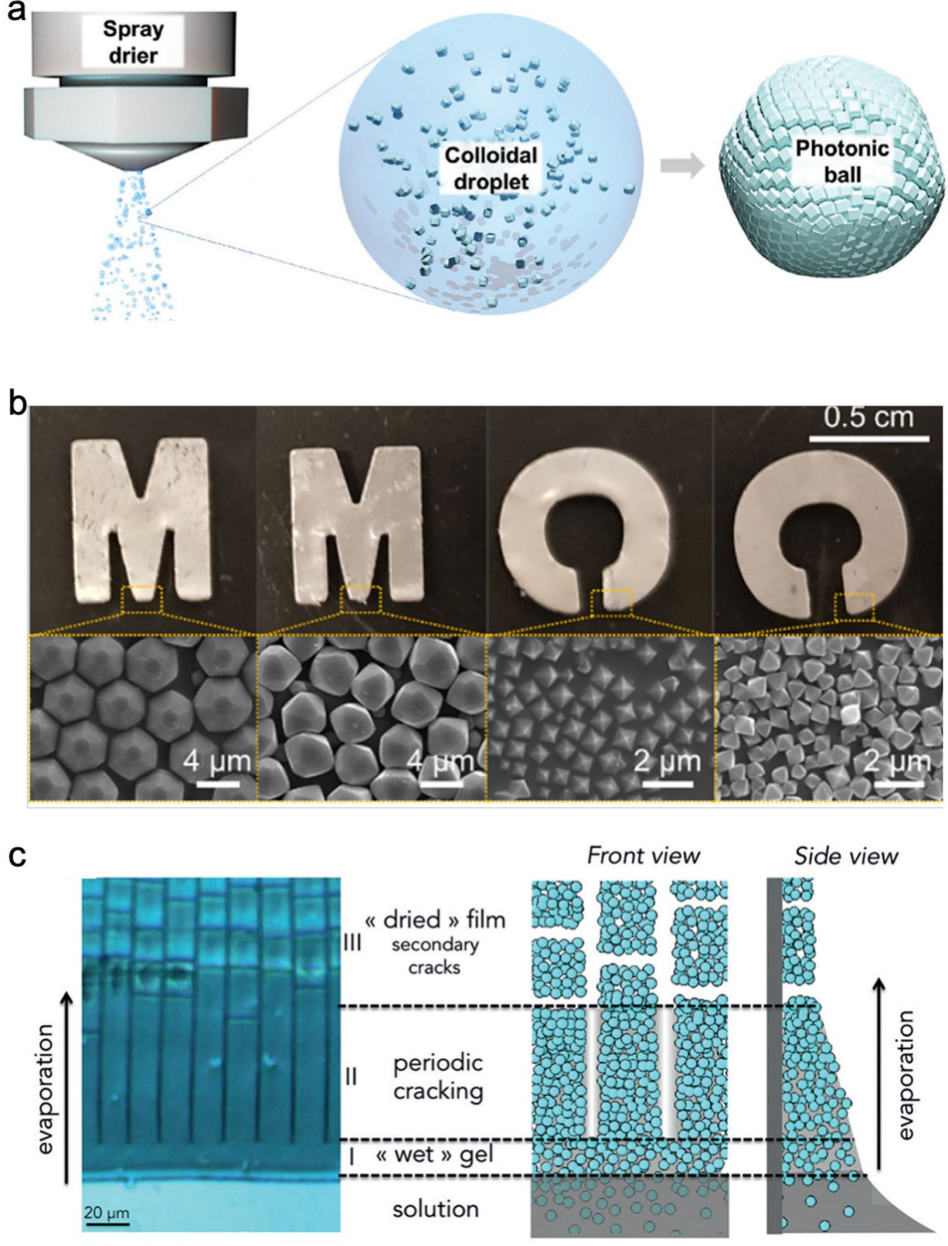
a) Schematic illustration of the self-assembly of ZIF-8 particles via spray drying. Figure 7a was reproduced from [138], C. Avci et al., “Metal-Organic Framework Photonic Balls: Single Object Analysis for Local Thermal Probing”, Advanced Materials, with permission from John Wiley and Sons. Copyright © 2021 WILEY‐VCH GmbH. This content is not subject to CC BY 4.0. b) Photos of aligned MIL96−PDMS films on shaped aluminum foil. Figure 7b was reprinted with permission from [139], Copyright 2021, American Chemical Society. This content is not subject to CC BY 4.0. c) Optical micrograph and schematic illustration of the evaporation-directed with assembly with periodic cracks. Figure 7c was reproduced from [141], O. Dalstein et al., “Evaporation-Directed Crack-Patterning of Metal-Organic Framework Colloidal Films and Their Application as Photonic Sensors”, Angewandte Chemie International Edition, with permission from John Wiley and Sons. Copyright © 2017 Wiley-VCH Verlag GmbH & Co. KGaA, Weinheim. This content is not subject to CC BY 4.0.

One problem regarding the assembly of monocrystalline coordination polymers is that interparticle voids always exist among the self-assembled polyhedral particles because of steric effects and the repulsion–attraction equilibrium among the particles. Even with the assistance of a gradient centrifugation field, the crystalline nanocubes still could not be packed into void-free structures [137]. The unavoidable interparticle voids limit interparticle interactions among the crystals, making the assemblies difficult to control. To solve this problem, assembling was tried in confined spaces, such as droplets, liquid–liquid interfaces, on the surface of substrates, or under a gradient centrifugation field. Spray dry could confine the colloidal particles in droplets. During evaporation, the particles could be squeezed through the shrinkage of the droplet size (Figure 7a) [138]. The polyhedra were assembled into balls, which could be used for measuring the temperature. In some parts of the photonic balls, the particles were attached tightly. However, even in this case, voids still could not be avoided. Monocrystalline coordination polymers were also dispersed in a solvent containing a polydimethylsiloxane (Figure 7b) [139]. The mixed solution was cast at liquid–liquid or air–liquid interfaces. After evaporation of the solvent, the monocrystalline coordination polymers were packed by the condensed polymer, forming free-standing films. The coordination polymers could also be grown on 1D polyimide fibers first [140]. Then, the 1D fibers could self-assemble into networks, which could simultaneously provide mechanical strength and continuous pathways for ions. These networks worked well as solid-state electrolytes in Li-ion batteries. To assemble the coordination polymer particles into a 2D configuration, dip-coating deposition was employed [141]. The evaporation of solvent continuously arranged the particles. However, due to a non-uniform distribution of the suspension on the surface and the difference in local evaporation and flow condition, cracks could form between micrometer-sized domains (Figure 7c) [141]. The crack propagation and periodicity could be tailored by controlling the evaporation front and the withdrawal speed, making photonic sensors possible. To pack the coordination polymer particles denser, stronger forces were introduced by casting the particle dispersion at an ice–water interface [142]. After freezing of the residual water, the particles were squeezed by ice in a 2D space, forming nanosheets.

2D monocrystalline coordination polymers are anisotropic and, thus, very suitable to enhance inter-crystal interaction by forming laminar stacking superstructures. This concept was earlier demonstrated by applying a solution-based layer-by-layer growth technique coupled with the Langmuir–Blodgett method [143]. Both out-of-plane and in-plane orientations could be realized by using only two simple components (free-base porphyrin molecular building blocks and metal-ion joints without using pillaring units), suggesting the possibility to assemble 2D coordination polymers. To facilitate the assembly of 2D coordination polymers, a modular assembly strategy was developed [144–145]. In the process, the hydrophobic 2D coordination polymers nanosheets were dispersed in ethanol first. Then, the suspension was spread on the surface of water. The nanosheets spontaneously and precisely organized into films at the interface laminarly. The films could be easily transferred to other substrates, and showed superprotonic conductivity, which may be promising for fuel cells.

**Figure 8:**
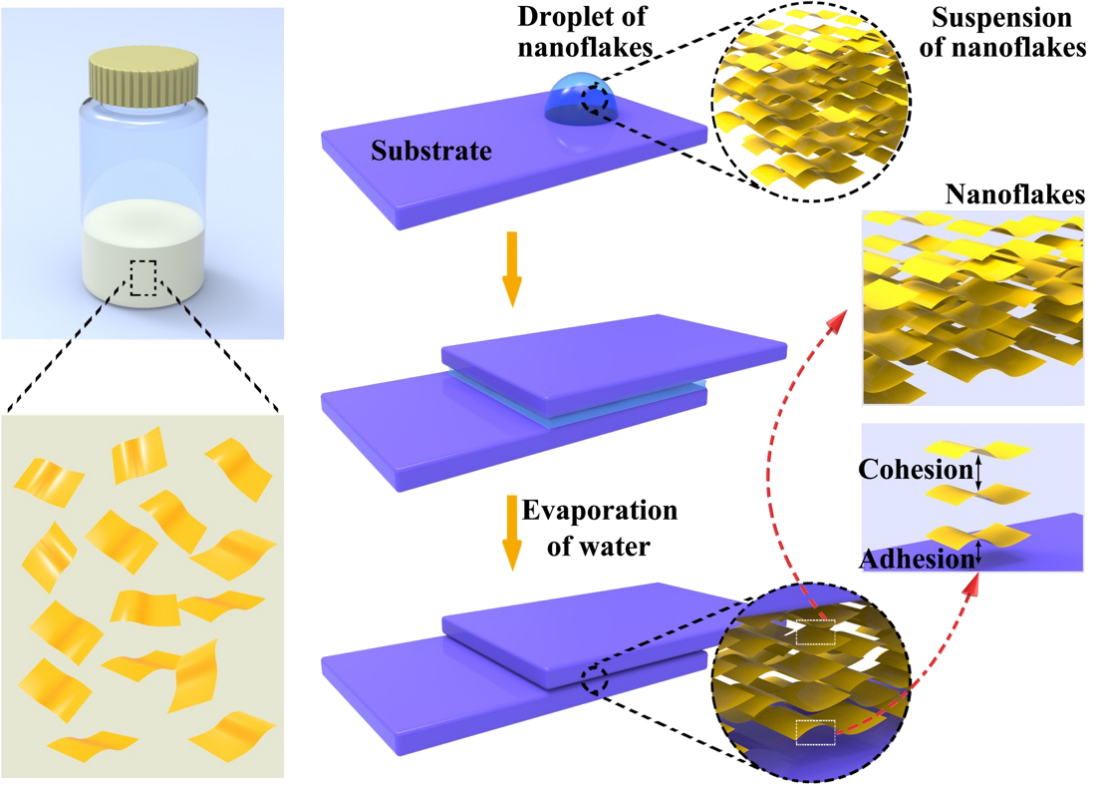
Illustration of packing nanoflakes between substrates through evaporation of a dispersion of nanoflakes. Figure 8 was reprinted with permission from [147], Copyright 2017, American Chemical Society. This content is not subject to CC BY 4.0.

The assembly process of 2D coordination polymer nanosheets could be confined inside the space between sandwiched substrates [146–147]. Evaporation of water could be confined at the edges of the suspension, forming local flows to arrange the nanoflakes parallelly. As a result, the dried nanoflakes stacked laminarly, working as glue to bond the glass slides (Figure 8) [147]. The shear adhesion strength could reach about 50 N·cm^−2^ for different kinds of substrates. The stacking nanoflakes formed multiple domains. Most of the domains were quasi-parallel to the substrates, suggesting a correlation between the alignment of nanoflakes and the anisotropic adhesion strength. The adhesion function is quite attractive because this function is versatile even for nanoparticles. Electrodes of sodium-ion batteries can be fabricated by using the Ni–CN–Ni colloids as glue.

**Figure 9:**
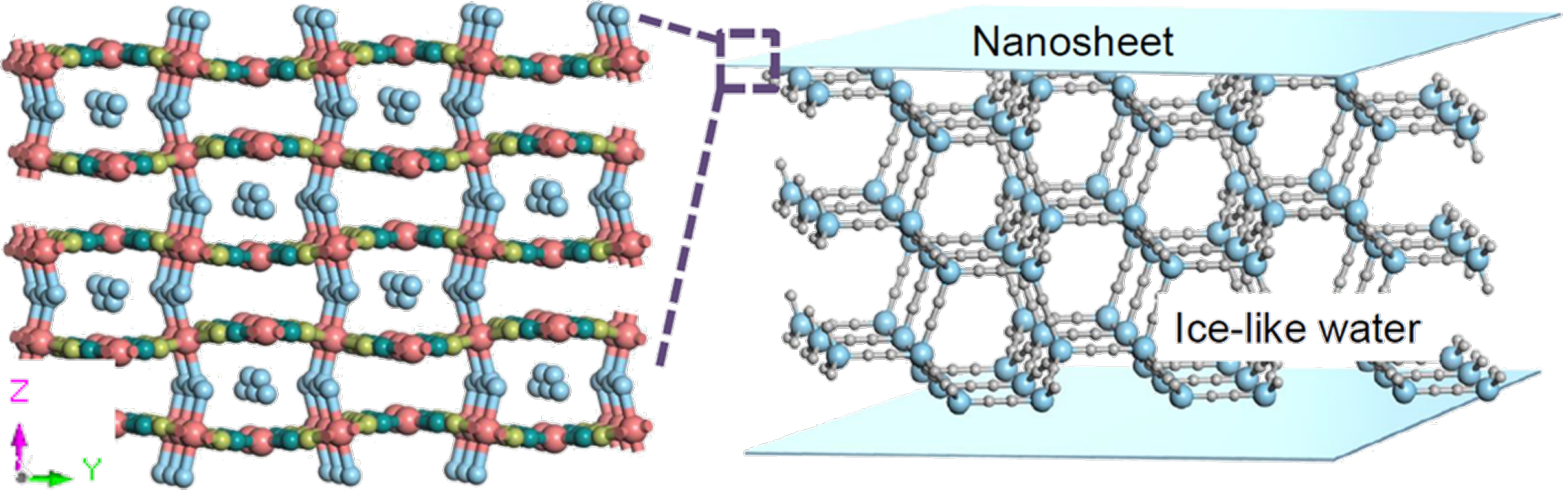
Illustration of the possible structure of water inside the Ni–CN–Ni nanosheets. The green balls represent carbon. The red balls represent nickel. The yellow balls represent nitrogen. The blue balls represent oxygen. The gray balls represent hydrogen. Figure 9 was used with permission of The Royal Society of Chemistry from [149] (“An X-state solid–liquid mixture with unusual mechanical properties formed by water and coordination polymer nanosheet nanoarchitectonics” by C. Shi et al., Nanoscale, vol. 14, issue 20, © 2022); permission conveyed through Copyright Clearance Center, Inc. This content is not subject to CC BY 4.0.

The contribution to the adhesion strength among 2D coordination polymers was generally considered to be van der Waals forces [147]. However, the adhesion strength varied over time, sometimes reaching values even close to 100 N·cm^−2^, which is higher than to be expected from van der Waals forces. The non-uniform deposition of Ni–CN–Ni nanosheets caused by Marangoni flow was an important reason for the unstable value of the adhesion strength [148]. Therefore, doughs with less liquid fraction were used to minimize the influence of the colloidal Marangoni flow. The shear adhesion strengths were increased to ca. 200 N·cm^−2^. Such a high value indicated contributions from other forces. By carefully investigating the water content and the infrared spectrum, hydrogen bonding between the nanosheets and the ice-like water molecules were found to be the main contributors to the unique adhesion behavior (Figure 9) [149]. This finding suggests that the 2D coordination polymers could cooperatively assemble into useful macrostructures with considerable mechanical strength. For instance, DUT-134(Cu) plates could be assembled into carpets and tubes through face-to-face or edge-to-face alignment [150]. The carpets and tubes showed excellent performance as air filter. The uptake of H_2_S by the carpets is 6–7 times higher than that of bulk crystals. The interesting part of the unique adhesion property is not only to bring new adhesives, but also the opportunity to provide stable assemblies, which can be recognized as colloids in extreme states. These colloids contain water and 2D nanosheets, and the water molecules and the 2D nanosheets are confined by each other. The electronic structures of the molecules in the colloids should be strongly alternated, leading to functional solids.

## Conclusion

In this review, we introduced recent advances in nanoarchitectures of monocrystalline coordination polymers through confined assembly. The related work was summarized into two main categories, namely confined crystallization and confined assembly of monocrystalline coordination polymers. When the crystallization of monocrystalline coordination polymers happened inside the networks, the process could be recognized as a filling process of the lattices in the connected vessels in the networks. The front of the filled lattices could move along the connected vessels, occupying several branches or even swallowing the networks. These facts eventually led to three kinds of nanoarchitectures of coordination polymers: networks@crystal, ‘networks+molecules’@crystal, and micro–meso–macroporous crystals. The physiochemical properties of the nanoarchitectures were changed significantly compared with pure coordination polymers, making the coordination polymer suitable for applications such as batteries, sensors, biomedical applications. When the assembly of monocrystalline coordination polymers particles happened inside a confined space, anisotropic ordering of the particles emerged. Confined assembly was of significant importance for 2D-shaped coordination polymers because the ordered assembly of coordination polymers was usually hindered by steric effects. Confined environments offer geometrical confinement and local flow with shear forces, which were particularly effective for generating laminar stacking structures. The packed coordination polymers showed unique mechanical properties and performed excellent in batteries and pollutant treatment.

This field brings new opportunities in research. From the point view of physics and chemistry, the interactions at the interfaces need to be clarified, and the kinetics under the influence of the confined environment are worth investigating. Particularly, additional concepts, such as photon-irradiation, mechanical deformation, or electrical and magnetic forces could influence the interactions and the formation kinetics of the interest structures. From the point view of materials, it would be interesting to use confined growth and assembly to assemble the coordination polymers that could satisfy practical requirements from engineers in specific areas such as batteries, biomedicine and water/air treatment. It would also be interesting to use the currently available products, because their performance in some specific applications is already very good now.

We should note that there still are some challenges to be tackled in the future. First, the detailed structures of the networks@crystal are difficult to investigate. The atomic structures of the composites are easily lost during observation because the coordination polymers are not stable under electron irradiation. Moreover, when the composites are within the length range from 1 to 10 μm, the whole structure of the composites cannot be imaged well by electron microscopy or X-ray based imaging techniques. Second, time-resolved structural changes of the nanoarchitectonics are hard to be observed because of the complex processing environment. However, the time-resolved information is essential for establishing reliable mechanisms to interpret and control the formation process during the confined assembly.
